# Stability analysis of a neural field self-organizing map

**DOI:** 10.1186/s13408-020-00097-6

**Published:** 2020-12-01

**Authors:** Georgios Detorakis, Antoine Chaillet, Nicolas P. Rougier

**Affiliations:** 1 adNomus Inc., San Jose, CA USA; 2 CentraleSupélec, Laboratoire des Signaux et Systèmes, Université Paris Saclay, Gif-sur-Yvette, France; 3 Institut Universitaire de France, Paris, France; 4 Inria Bordeaux Sud-Ouest, Bordeaux, France; 5 Institut des maladies neurodégénératives, CNRS, Université de Bordeaux, Bordeaux, France

**Keywords:** Self-organizing maps, Neural fields, Lyapunov function, Asymptotic stability, Neural networks

## Abstract

We provide theoretical conditions guaranteeing that a self-organizing map efficiently develops representations of the input space. The study relies on a neural field model of spatiotemporal activity in area 3b of the primary somatosensory cortex. We rely on Lyapunov’s theory for neural fields to derive theoretical conditions for stability. We verify the theoretical conditions by numerical experiments. The analysis highlights the key role played by the balance between excitation and inhibition of lateral synaptic coupling and the strength of synaptic gains in the formation and maintenance of self-organizing maps.

## Introduction

Self-organizing maps (SOMs) are neural networks mapping a high-dimensional space to a low-dimensional one through unsupervised learning. They were first introduced by Grossberg [14] and later by Kohonen [19]. SOMs are widely used in computer science and data analysis for quantization and visualization of high-dimensional data [25, 38]. They also constitute a suitable tool in computational neuroscience to study the formation and maintenance of topographic maps in primary sensory cortices such as the visual cortex [24, 31] and the somatosensory cortex [13, 34]. Many variations and applications of Kohonen’s SOM algorithm can be found in [16] and [27].

A type of self-organizing map based on neural fields theory has been introduced in [8], where neural fields are used to drive the self-organizing process. Neural fields are integrodifferential equations that describe the spatiotemporal dynamics of a cortical sheet [3–5]. The SOM proposed in [8] describes the topographic organization of area 3b of the primary somatosensory cortex of monkeys [21, 28]. The model relies on an earlier work [29] known as the dynamic SOM (DSOM) algorithm. DSOM provides an online SOM learning algorithm, where the Kohonen’s SOM time-dependent learning rate and neighborhood function have been replaced by time-invariant ones. The DSOM neighborhood function and learning rate solely depend on the distance of the winner unit (i.e., the most active neuron) from the input. The model proposed in [8, 9] combines the DSOM time-invariant learning rate and neighborhood function with Oja’s learning rule [26]. As thoroughly described in [8, 9], the model is compatible with anatomical evidence of how area 3b in monkeys develops, maintains, and reorganizes topographic representations of a skin patch of the index finger.

In this work, we provide theoretical insights on the stability and convergence of the neural field SOM algorithm proposed in [8, 9] by studying a more general class of systems than that originally proposed in [8]. We use Lyapunov’s stability theory adapted to neural field dynamics [11]. Since typical activation functions employed in the model (such as absolute values or rectification functions) are not necessarily differentiable, we do not rely on linearization techniques but rather directly assess the stability of the original nonlinear dynamics. Yet, the obtained results are local, meaning that they are valid only for initial conditions in the vicinity of the considered equilibrium. Nonetheless, we show that they agree with numerical simulations. The stability conditions derived in this work can be used toward the direction of tuning neural field models such that they achieve the best possible results in developing self-organizing maps and thus more generalized representations. Moreover, the conditions we propose indicate that the balance between lateral excitation and inhibition keeps the system stable, thus ruling out possible configurations in which learning does not take place properly. These findings are in line with both experimental observations [18, 30] and computational modeling [35–37].

The paper is organized as follows. In Sect. 2, we recall the SOM model under concern and its basic mechanisms. In Sect. 3, we present our main theoretical results, which we confront to numerical simulations in Sect. 4. A discussion on the obtained results is provided in Sect. 5. Mathematical proofs are given in Sect. 6.

## Self-organizing neural fields

### Neural population dynamics

We consider the following neural fields equation:

1 $$\begin{aligned} \tau \frac{\partial u}{\partial t}(r,t) &= -u(r,t) + \int _{\Omega }w_{l}\bigl( \bigl\vert r- r' \bigr\vert \bigr)\operatorname{rect}\bigl(u\bigl(r',t\bigr)\bigr)\,dr'+ I, \end{aligned}$$

where Ω is a connected compact subset of $\mathbb {R}^{q}$ ($q=1,2,3$). For $q=2$, the integral of a function $g:\Omega =\Omega _{1}\times \Omega _{2}\to \mathbb{R}$ is to be understood as $\int _{\Omega }g(r)\,dr=\int _{\Omega _{1}}\int _{\Omega _{2}} g(r_{1},r_{2})\,dr_{2}\,dr_{1}$ with $r=(r_{1},r_{2})$, and similarly for $q=3$; $u(r,t)$ represents the mean membrane potential at position $r\in \Omega $ and time $t\geq 0$, *τ* is a positive decay time constant, *I* denotes an external input, and $w_{l}$ is a function that represents the strength of lateral synaptic coupling. It is given by

2 $$\begin{aligned} w_{l}(x)= w_{e}(x) - w_{i}(x), \end{aligned}$$

where the excitation and inhibition synaptic weights are typically given by

3a $$\begin{aligned} &w_{e}(x)=K_{e} e^{-x^{2}/2\sigma _{e}^{2}} \end{aligned}$$

and

3b $$\begin{aligned} &w_{i}(x)=K_{i} e^{-x^{2}/2\sigma _{i}^{2}} \end{aligned}$$

with $K_{e},K_{i},\sigma _{e},\sigma _{i}>0$. In [8, 9] the input is provided through a two-dimensional skin model. The skin model is composed of a two-dimensional grid and receptors. The receptors are points distributed on the surface of the grid (uniformly). When a stimulus is applied on the grid, the receptors sample the input signal and convey the information to the cortical model. The skin stimulus is a noisy Gaussian-like function, and the input to the neural fields model is provided by the following function:

4 $$\begin{aligned} I(r,p,t) &= 1 - \frac{ \vert w_{f}(r,t)-s(p) \vert _{1}}{m}, \end{aligned}$$

where $|\cdot |_{1}$ denotes the 1-norm: $|x|_{1}=\sum_{i=1}^{m} |x_{i}|$, and $s:\mathbb{R}^{2} \rightarrow [0,1]^{m}$ is a function that maps the raw input from the two-dimensional skin space to $[0,1]^{m}$. For instance, for a tactile stimulus at position $p\in \mathbb {R}^{2}$ on the skin, $s(p)\in \mathbb {R}^{m}$ could be defined as the normalized distance from *p* to the location of each receptor, thus potentially of much higher dimension than 2. For a more detailed description of receptor model, see [9]. The function $w_{f}: \Omega \times \mathbb {R}_{\geq 0}\to \mathbb {R}^{m}$ represents feed-forward synaptic weights with value updated according to

5 $$\begin{aligned} \frac{\partial w_{f}}{\partial t}(r,t) &= \gamma \bigl( s(p) - w_{f}(r,t) \bigr) \int _{\Omega }w_{e}\bigl( \bigl\vert r- r' \bigr\vert \bigr)\operatorname{rect}\bigl(u\bigl(r',t\bigr)\bigr)\,dr', \end{aligned}$$

where *γ* is a positive constant that represents the learning rate, and $\operatorname{rect}(x) = \max \{x, 0\}$. It is worth observing that since $s(p)\in [0,1]^{m}$, $w_{f}(r,t)\in [0,1]^{m}$ for all $r\in \Omega $ and $t\geq 0$ given any initial conditions satisfying $w_{f}(r,0)\in [0,1]^{m}$ for all $r\in \Omega $ (this can be seen by observing that the entries of $\frac{\partial w_{f}}{\partial t}(r,t)$ are negative as soon as the corresponding entries of $w_{f}(r,t)$ become greater than 1; similarly, they are positive when the corresponding entries of $w_{f}(r,t)$ get below 0: see (5)). Hence $\frac{|w_{f}(r,t)-s(p)|_{1}}{m}\in [0,1]$ at all times. Thus expression (4) can be interpreted as a high input when the feedforward weights are close to $s(p)$ and as a lower input when these are more distant.

The overall model (1), (4), (5) reflects the dynamics of a cortical neural population in combination with a learning rule of the feed-forward connections $w_{f}$, which convey information from receptors to the cortical sheet. As described in [8, 9], this model can express a variety of different behaviors, depending on the lateral connectivity kernels $w_{e}$ and $w_{i}$.

The main advantage of the learning rule given by Eq. (5) is that it is a biologically plausible modification of the DSOM learning rule [29]. In DSOM the learning rate and neighborhood function are time-invariant and can adapt to the input according to one single parameter, called elasticity. This particular modification leads to the following behavior: if the winner neuron (i.e., the neuron that has the shortest distance from the input stimulus to its corresponding codebook–weight) is close to the stimulus, then the neighborhood function shrinks around it. This results in making the weights of neurons within the dynamic neighborhood stronger and the weights of the other units weaker. However, when the winning unit is very far from the current input, the neighborhood function exhibits a broad activity pattern, promoting learning of every unit in the network. Therefore in [8] the neighborhood function has been replaced by the term $\int _{\Omega }w_{e}(|r-r'|)\operatorname{rect}(u(r',t))\,dr'$, providing a more realistic and biological plausible learning algorithm for self-organizing maps in the context of neuroscience.

### Self-organizing maps

**Figure 1:**
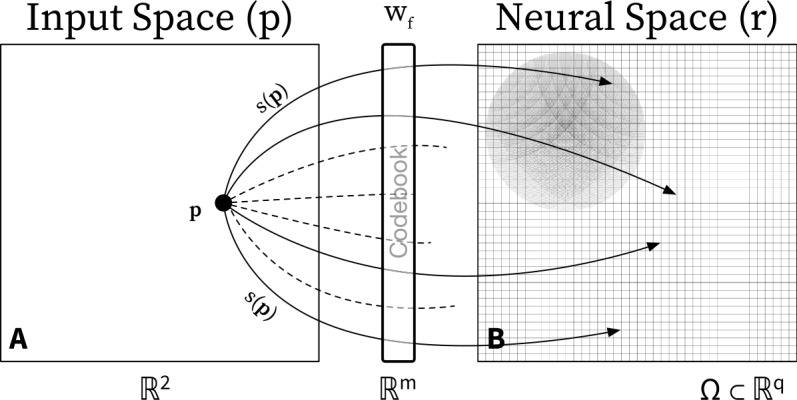
Neural field self-organizing map. Graphical representation of the learning algorithm introduced in [8, 9]. (**A**) Tactile bidimensional stimuli are mapped to an *m*-dimensional space through a function *s* that involves the skin receptors. This function is used to update the codebooks, which are then mapped to the neural space Ω of lower dimension. The input *I* receives the mapped input sample and provides input to the neural field and codebook equations. (**B**) The numerical steady-state solution of Eq. (1) (i.e., bump) defines the neighborhood (the group of neurons) that will have its neurons updating their codebooks based on Eq. (5)

We start by briefly describing how the SOM model introduced in [8] and [9] works. The algorithm starts by initializing the feed-forward weights randomly (usually uniformly), and the neural field activity $u(r, 0)$ is set to zero. The second step is sampling the input space by randomly drawn samples of dimension *m* from an input distribution. At every epoch, one sample is given to the neural field (1) and (5) through Eq. (4). This first step is depicted in Fig. 1(A), where a two-dimensional point $p=(p_{1}, p_{2})$ is sampled from a uniform distribution, $p_{1}, p_{2} \sim \mathcal{U}(0, 1)$. The samples are mapped to the neural space through the function *s* and then are passed to Eq. (4). At this point, we should point out that there are two ways of presenting stimuli while training a self-organizing map. The first is predetermining an amount of input samples and present one at each epoch (online learning) and the second is collecting all the input samples into a batch and giving all of them at once to the network (batch learning). In this work, we use the former (online learning) since it is biologically plausible.

Then the algorithm proceeds with computing the numerical solution of Eqs. (1) and (5). To that aim, Eqs. (1) and (5) are discretized and solved numerically using Euler’s forward method. The numerical solution of Eq. (1) is typically a bell-shaped curve (bump) centered on the neuron that is the closest unit to the input sample and therefore is called the winner neuron or best matching unit (BMU). In Fig. 1(B), this is depicted as a black disc on a discrete lattice. The lattice represents a discretization of the field where each tile corresponds to a neuron. Neurons that lie within the vicinity (within the black disc in Fig. 1(B)) defined by the solution of Eq. (1) update their weights based on Eq. (5). The rest of the neurons feed-forward weights remain in their previous state. Once the temporal integration of Eqs. (1) and (5) is complete, the activity of the field is reset to its baseline activity. Then another input sample is drawn, and the whole process repeats itself. Once the number of epochs is exhausted, the learning stops, and the mapping process is completed.

To make the aforementioned algorithm directly comparable to Kohonen SOM [19], we provide some insights. First, in Kohonen’s SOM, we compute the distance between the input and the codebooks. Here we do the same using Eq. (4). The neighborhood function that Kohonen’s SOM uses to update the feed-forward weights is replaced here by the numerical solution of the neural field (Eq. (1)) and more precisely by the term $\int _{\Omega }w_{e}(|r-r'|)\operatorname{rect}(u(r', t))\,dr'$. Both the learning rate and the width of the neighborhood function are time-independent in our case, as opposed to Kohonen’s SOM, where they are both time-dependent. Our learning rule is different since we use a modified Oja rule [26], which is based on Hebbian learning [15], and it is therefore biologically plausible [1]. The dimensionality reduction in both models, the Kohonen and ours, takes place at the level of the learning rule. This means that Eq. (5) is responsible for learning the representations and mapping the input distribution (of dimensions *m*) on a manifold of lower dimension $q\in \{1,2,3\}$.

## Explicit conditions for stability

The most important question when one trains a self-organizing map is: *Will the learning process converge and properly map the input space to the neural one?* In most of the cases, it is not possible to predict this. However, in the specific case of the self-organizing algorithm provided by [8], here we show that it is possible to obtain an analytical condition that guarantees the stability of the equilibrium point of system (1)–(5). Stability during learning is a prerequisite to generate a meaningful mapping and thus a proper topographic map. Moreover, a byproduct of deriving such a stability condition is providing some insights on how to properly tune model parameters.

To this end, we now proceed to the mathematical analysis of the model. For generality, the adopted mathematical framework is slightly wider than merely Eqs. (1), (4), (5) and encompasses more general classes of activation functions and synaptic kernels. We start by introducing the considered class of systems and then provide sufficient conditions for its stability and convergence.

### Model under study

The self-organizing neural field (1), (4), (5) is a particular case of the more general dynamics

6a $$\begin{aligned} &\tau \frac{\partial u}{\partial t}(r,t)= -u(r,t)+ \int _{\Omega }w_{l}\bigl(r,r' \bigr)f_{l}\bigl(u\bigl(r',t\bigr) \bigr)\,dr'+ f_{s}\bigl(w_{f}(r,t)-s(p) \bigr), \end{aligned}$$

6b $$\begin{aligned} &\frac{\partial w_{f}}{\partial t}(r,t)= \gamma \bigl(s(p)-w_{f}(r,t) \bigr) \int _{\Omega }w_{e}\bigl(r,r' \bigr)f_{e}\bigl(u\bigl(r',t\bigr) \bigr)\,dr', \end{aligned}$$

where $\tau ,\gamma >0$, $w_{l},w_{e}\in L_{2}(\Omega ^{2},\mathbb{R})$, the set of all square-integrable functions from $\Omega ^{2}$ to $\mathbb{R}$, and $f_{e}$, $f_{l}$ and $f_{s}$ are Lipschitz continuous functions.

### Existence of equilibrium patterns

Assuming that $\inf_{r\in \Omega }\int _{\Omega } w_{e}(r,r') f_{e}({u^{*}}( r'))\,dr'\neq 0$, any equilibrium pattern $({u^{*}},w_{f}^{*})$ of (6a) and (6b) satisfies the following equations:

7a $$\begin{aligned} &u^{*}(r)= f_{s}(0)+ \int _{\Omega }w_{l}\bigl(r,r' \bigr)f_{l}\bigl(u^{*}\bigl(r'\bigr) \bigr)\,dr', \end{aligned}$$

7b $$\begin{aligned} &w_{f}^{*}(r)= s(p). \end{aligned}$$

Since $\omega _{l}\in L_{2}(\Omega ^{2},\mathbb{R})$, [12, Theorem 3.6] ensures the existence of at least one such equilibrium pattern.

### Stability analysis of Eq. (6a) and (6b)

We recall that an equilibrium $x^{*}$ of a system $\dot{x}(t)=f(x(t))$, where $x(t):\Omega \to \mathbb {R}^{n}$ for each fixed $t\geq 0$, is called *globally exponentially stable* if there exist $k,\varepsilon >0$ such that, for all admissible initial conditions,

8 $$\begin{aligned} \bigl\Vert x(t)-x^{*} \bigr\Vert \leq k \bigl\Vert x(0)-x^{*} \bigr\Vert e^{-\varepsilon t},\quad \forall t \geq 0, \end{aligned}$$

where $\|\cdot \|$ denotes the spatial $L_{2}$-norm. This property ensures that all solutions go to the equilibrium configuration $x^{*}$ in the $L_{2}$ sense (global convergence) and that the transient overshoot is proportional to the $L_{2}$-norm of the distance between the initial configuration and the equilibrium (stability). The equilibrium pattern $x^{*}$ is said to be *locally exponentially stable* if (8) holds only for solutions starting sufficiently near from it (in the $L_{2}$ sense). We refer the reader to [11] for a deeper discussion on the stability analysis of neural fields.

Our main result proposes a sufficient condition for the local exponential stability of Eq. (6a) and (6b). Its proof is given in Sect. 6.1.

#### Theorem 1

*Let* Ω *be a compact connected set of* $\mathbb {R}^{q}$, *let* $w_{l}\in L_{2}(\Omega ^{2},\mathbb{R})$, *and let* $w_{e}:\Omega ^{2}\to \mathbb {R}$ *be a bounded function*. *Assume further that* $f_{l}$, $f_{s}$, *and* $f_{e}$ *are Lipschitz continuous functions*, *and let* $\ell _{l}$ *denote the Lipschitz constant of* $f_{l}$. *Let* $(u^{*},w_{f}^{*})$ *denote any equilibrium of Eq*. (6a) *and* (6b), *as defined in Eq*. (7a) *and* (7b). *Then*, *under the conditions*

9 $$\begin{aligned} \sqrt{ \int _{\Omega } \int _{\Omega }w_{l}\bigl(r,r' \bigr)^{2}\,dr'\,dr}&< \frac{1}{\ell _{l}} \end{aligned}$$

*and*

10 $$\begin{aligned} \inf_{r\in \Omega } \int _{\Omega } w_{e}\bigl(r, r'\bigr) f_{e}\bigl({u^{*}}\bigl(r'\bigr)\bigr)\,dr'&>0, \end{aligned}$$

*the equilibrium pattern* $(u^{*},w_{f}^{*})$ *is locally exponentially stable for Eq*. (6a) *and* (6b).

Condition (9) imposes that the synaptic weights of the lateral coupling $w_{l}$ are sufficiently small: stronger lateral synaptic weights can be tolerated if the maximum slope ${\ell_{l}}$ of the activation function $f_{l}$ is low enough, meaning that the system given by Eq. (6a) is less self-excitable. Recall that if $f_{l}$ is a differentiable function, then $\ell _{l}$ can be picked as the maximum value of its derivative. Nonetheless, Theorem 1 does not impose such a differentiability requirement, thus allowing us to consider nonsmooth functions such as absolute values, saturations, or rectification functions. Note that it was shown in [33] that condition (9) ensures that the system owns a single equilibrium pattern. It is also worth stressing that the slopes of the functions $f_{s}$ and $f_{e}$ do not intervene in the stability conditions.

Condition (10) requires a sufficient excitation in the vicinity of the equilibrium $u^{*}$. Roughly speaking, it imposes that the considered equilibrium pattern $u^{*}$ does not lie in a region where $f_{e}$ is zero.

### Stability analysis of the SOM neural fields

Theorem 1 provides a stability condition for the model described by Eq. (6a) and (6b). We next apply it to the model given in [8] to derive more explicit and testable stability conditions. More precisely, the self-organizing neural fields (1), (4), (5) can be put in the form of Eq. (6a) and (6b) by letting $f_{e}(x)=f_{l}(x)=\text{rect}(x)$, $f_{s}(x)= 1-\frac{|x|_{1}}{m}$, and

11a $$\begin{aligned} &w_{e}\bigl(r,r'\bigr)=K_{e} e^{-|r-r'|^{2}/2\sigma _{e}^{2}}, \end{aligned}$$

11b $$\begin{aligned} &w_{i}\bigl(r,r'\bigr)=K_{i} e^{-|r-r'|^{2}/2\sigma _{i}^{2}}, \end{aligned}$$

11c $$\begin{aligned} &w_{l}\bigl(r,r'\bigr)=w_{e} \bigl(r,r'\bigr)-w_{i}\bigl(r,r'\bigr). \end{aligned}$$

In view of (7a) and (7b), the equilibrium patterns of Eqs. (1), (4), (5) are given by

12a $$\begin{aligned} &u^{*}(r)= 1+ \int _{\Omega }w_{l}\bigl(r,r'\bigr) \operatorname{rect}\bigl(u^{*}\bigl(r'\bigr) \bigr)\,dr', \end{aligned}$$

12b $$\begin{aligned} &w_{f}^{*}(r)= s(p). \end{aligned}$$

The Lipschitz constant of $f_{l}$ is $\ell _{l}=1$. Based on this, we can also derive the following corollary, whose proof is provided in Sect. 6.2.

#### Corollary 1

*Assume that* Ω *is a compact connected set of* $\mathbb{R}^{q}$, *and let* $w_{e}$, $w_{i}$, *and* $w_{l}$ *be as in* (11a)*–*(11c). *Then*, *under the condition that*

13 $$\begin{aligned} \int _{\Omega } \int _{\Omega } \bigl(K_{e} e^{-|r-r'|^{2}/2\sigma _{e}^{2}}-K_{i} e^{-|r-r'|^{2}/2\sigma _{i}^{2}} \bigr)^{2}\,dr'\,dr< 1, \end{aligned}$$

*the equilibrium* $(u^{*},w_{f}^{*})$, *as defined in Eq*. (12a) *and* (12b), *is locally exponentially stable for Eqs*. (1)*–*(5).

A particular case for which local exponential stability holds is when the excitation and inhibition weight functions are sufficiently balanced. Indeed, it appears clearly that Eq. (13) is fulfilled if $K_{e}\simeq K_{i}$ and $\sigma _{e}\simeq \sigma _{i}$. See the discussion in Sect. 5 for further physiological insights on this condition.

The integral involved in (13) can be solved explicitly. For instance, in the two-dimensional case ($q=2$) the condition boils down to the following:

#### Corollary 2

*Let* $\Omega =[a,b]\times [a,b]$ *for some* $a,b\in \mathbb{R}$ *with* $b\geq a$, *and let* $w_{e}$, $w_{i}$, *and* $w_{l}$ *be as in* (11a)*–*(11c). *Define*

14 $$\begin{aligned} \xi _{a,b}(\sigma ):= \biggl(2\sigma ^{2} \bigl(e^{- \frac{(a-b)^{2}}{2\sigma ^{2}}}-1 \bigr)+\sigma \sqrt{2\pi }(a-b) \operatorname{Erf} \biggl(\frac{a-b}{\sigma \sqrt{2}} \biggr) \biggr)^{2},\quad \forall \sigma >0, \end{aligned}$$

*where* $\operatorname{Erf}:\mathbb{R}\to (-1,1)$ *denotes the Gauss error function*. *Then*, *under the condition*

15 $$\begin{aligned} K_{e}^{2}\xi _{a,b}(\sigma _{e}/\sqrt{2})+K_{i}^{2}\xi _{a,b}(\sigma _{i}/ \sqrt{2})-2K_{e}K_{i} \xi _{a,b} \biggl( \frac{\sigma _{e}\sigma _{i}}{\sqrt{\sigma _{e}^{2}+\sigma _{i}^{2}}} \biggr)< 1, \end{aligned}$$

*the equilibrium* $(u^{*},w_{f}^{*})$, *as defined in Eq*. (12a) *and* (12b), *is locally exponentially stable for Eq*. (1)*–*(5).

Plenty of approximations are available for the Erf function in the literature. For instance, the following expression approximates it with a 5.10^−4^ error:

$$\begin{aligned} \operatorname{Erf}(x)\simeq 1- \frac{1}{(1+a_{1}x+a_{2}x^{2}+a_{3}x^{3}+a_{4}x^{4})^{4}} \end{aligned}$$

with $a_{1} = 0.278393$, $a_{2} = 0.230389$, $a_{3} = 0.000972$, and $a_{4} = 0.078108$; see, for instance, [2]. The Erf function is also commonly implemented in mathematical software, thus making Eq. (15) easily testable in practice.

## Numerical assessment on a two-dimensional map

To numerically assess whether the above stability condition correctly predicts the performance of the learning process, we focus on a simple example of a two-dimensional map ($q=2$) and a two-dimensional input space ($n=2$). Furthermore, we choose $s(p)$ to be the identity function since we do not consider any receptors: the position of the tactile stimuli is assumed to be directly available. This choice is motivated by the fact that the presence or absence of a receptors grid does not affect the theoretical results of the current work. We refer to [8, 9] for a more complex application of the neural field self-organizing algorithm.

We sample two-dimensional inputs from a uniform distribution. Therefore we have $s_{i}(p) = (p_{1}, p_{2})$, where *i* indicates the *i*th sample, and $p_{1}, p_{2} \sim \mathcal{U}(0, 1)$. In all our simulations, we use 7000 sample points and train the self-organizing map over each of them (7000 epochs). It is worth stressing the difference between the training time (epochs) and the simulation time. The former refers to the iterations over all the input samples (stimuli): one such input is presented to the model at each epoch. The latter is attributed to the numerical temporal integration of Eqs. (1)–(5). Thus each epoch corresponds to a predefined number of simulation steps. At the end of each epoch the activity of the neural field is reset to baseline activity before proceeding to the next epoch.

### Parameters and simulation details

The neural fields equations are discretized using $k = 40\times 40$ units. Accordingly, the two-dimensional model (1)–(5) is simulated over a spatial uniform discretization $\Omega _{d}$ of the spatial domain $\Omega =[0,1]\times [0,1]$, namely $\Omega _{d}=\bigcup_{i,j=1}^{40} (\frac{i}{40},\frac{j}{40})$. The considered input space, over which the stimuli are uniformly distributed, is also $[0, 1]\times [0,1]$ (two-dimensional input vectors). The temporal integration is performed using the forward Euler method, whereas the spatial convolution in Eqs. (1)–(5) is computed via the fast Fourier transform (FFT). The learning process runs for 7000 epochs. The components of the feed-forward weights are initialized from a uniform distribution $\mathcal{U}(0, 0.01)$, and the neural field activity is set to zero. At each epoch, we feed a stimulus to Eqs. (1)–(5), and the system evolves according to its dynamics, whereas the feed-forward weights are being updated. Then we reset the neural fields activity to zero. We run each experiment ten times using a different pseudorandom number generator (PRNG) seed each time (the PRNG seeds are given in Appendix 7: the same initial conditions and the set of PRNG seeds were used in each experimental condition).

The source code is written in Python (Numpy-, Numba-, Sklearn, and Matplotlib-dependent) and are freely distributed under the GPL 3-Clause License (https://github.com/gdetor/som_stability). All the parameters used in numerical simulations are summarized in Table 1. All simulations ran on an Intel NUC machine equipped with an Intel i7-10th generation processor and 32 GB of physical memory, running Ubuntu Linux ($20.04.1$ LTS, Kernel: $5.4.0$-47-generic). The simulation of one self-organizing map consumes 493 MB of physical memory, and it took 2671 seconds to run the 7000 epochs.

**Table 1:** Simulation parameters. $K_{e}$ and $K_{i}$ are the amplitudes of excitatory and inhibitory lateral connections, respectively; $\sigma _{e}$ and $\sigma _{i}$ are the variances of excitatory and inhibitory lateral connections, respectively; *τ* is the decay time constant, *dt* is the integration time step in ms, *t* is the simulation time in seconds, and *γ* is the learning rate. In each epoch, one stimulus is presented to the model

	$K_{e}$	$\sigma _{e}$	$K_{i}$	$\sigma _{i}$	*τ*	*dt*	*t*	*γ*	epochs
Figure 2	0.90	0.11	0.86	1.0	1.0	0.015	25.0	0.002	7000
Figure 3	3.0	0.11	2.80	1.0	1.0	0.015	25.0	0.002	7000

### SOM quality measures

We measure the quality of the self-organizing maps using two performance indicators, the distortion $\mathcal{D}$ [6] and the $\delta x-\delta y$ representation [7]. We recall here that $\Omega _{d}$ is the spatial uniform discretization of $\Omega =[0, 1] \times [0, 1]$ and $k=40 \times 40$ is the number of nodes (neurons). Furthermore, for each $j\in \{1, \ldots , k\}$, $w^{j}_{f}(t^{*})$ denotes the steady-state value of the feed-forward weights at the *j*th node of the spatial discretization, and $t^{*}$ corresponds to the time at the end of an epoch.

The distortion assesses the quality of a self-organizing map. It measures the loss of information over the learning process. In other words, it indicates how good a reconstruction of an input will be after the mapping of all inputs to a lower-dimensional neural map. In a sense, distortion measures how well a SOM algorithm “compresses” the input data with respect to the neighborhood structure. Mathematically, the distortion is computed according to its discrete approximation:

16 $$\begin{aligned} \mathcal{D} &= \frac{1}{n} \sum _{i=1}^{n} \min _{j\in \{1, \ldots , k\}} \bigl\vert s_{i}(p) - w_{f}^{j} \bigl(t^{*}\bigr) \bigr\vert ^{2}, \end{aligned}$$

where *n* is the number of samples we use during the training of the self-organizing map.

Distortion is essentially an indicator of the map convergence, but it is not a reliable tool for assessing its quality. To gauge the quality of the map, we use the $\delta x - \delta y$ representation [7]. It shows when a map preserves the topology of the input space and hence how well a topographic map is formed. To estimate the $\delta x - \delta y$, we compute all the pairwise distances between the feed-forward weights, $\delta x = \delta x(i, j) = |w_{f}^{i}(t^{*}) - w_{f}^{j}(t^{*}) |$, and all the distances between the nodes of the uniform discretization of the input space $[0, 1]^{2}$, $\delta y(i, j) =|y_{i} - y_{j}|$ for $i, j = 1, \ldots , k$, where $y_{i}$ are the discrete nodes of $\Omega _{d}$. We plot the $\delta x - \delta y$ (i.e., *δx* is the ordinate, and *δy* the abscissa) along with a straight line, named $\mathcal{L}_{\delta x - \delta y}$, that crosses the origin and the mean of *δx* points. If the point cloud representation of $\delta x - \delta y$ closely follows the line $\mathcal{L}_{\delta x - \delta y}$, then the map is considered well-formed and preserves the topology of the input space.

To quantify the $\delta x - \delta y$ representation through a scalar performance index, we perform a linear regression on the point cloud of $\delta x - \delta y$ without fitting the intercept (magenta line in figures), and we get a new line named $\mathcal{L}_{\Delta }$. Then we define the measure $\mathcal{P} = \sqrt{\sum_{i=1}^{k}(a_{i} - b_{i})^{2}}$, where $a_{i} \in \mathcal{L}_{\delta _{x}-\delta y}$ and $b_{i} \in \mathcal{L}_{\Delta }$. Naturally, $\mathcal{P}$ should approach zero as the two lines are getting closer, indicating that the self-organizing map respects the topology of the input space, and thus it is well-formed.

### Stable case

**Figure 2:**
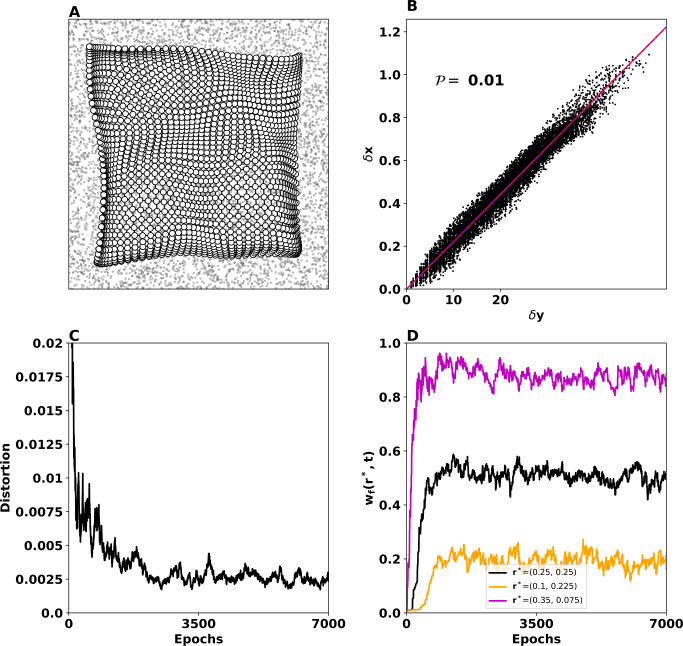
Two-dimensional SOM performance in the stable case. (**A**) Feed-forward weights (white discs) as they have been organized into a topographic map after 7000 epochs. The input in this case is a two-dimensional rectangular uniform distribution (black dots). (**B**) $\delta x - \delta y$ representation (black cloud), mean of *δx* (red line), and the linear regression of the $\delta x - \delta y$ representation (magenta line). The fact that the cloud is aligned around the red line indicates that the topographic map is well organized, as confirmed by a good index performance $\mathcal{P}=0.01$. (**C**) Distortion indicates that the loss of information during the learning process decreases, and the mapping of the input data to a two-dimensional self-organizing map respects the structure of the neighborhoods. (**D**) Temporal evolution of norm-2 of feed-forward weights of three neurons placed at $r^{\ast }=(0.25, 0.25)$, $(0.1, 0.225)$, and $(0.35, 0.075)$). Condition (15) is fulfilled, and therefore the weights converge to an equilibrium giving rise to a well-formed topographic map

We start by simulating the model described by Eqs. (1)–(5) with the parameters given in the first line of Table 1. With these parameters, condition (15) is fulfilled ($0.47 < 1$), and Corollary 2 predicts that the equilibrium is exponentially stable over each epoch. Accordingly, the model succeeds in building up a self-organizing map as shown in panel (A) of Fig. 2. The white discs indicate the feed-forward weights after learning, and the black dots indicate the input data points (two-dimensional rectangular uniform distribution).

Panels (B) and (C) show the $\delta x - \delta y$ representation and the distortion, respectively. We observe that the $\delta x - \delta y$ representation indicates a correlation between the feed-forward weights and the rectangular grid points (aligned with the mean of *δx*–red line). This means that the self-organizing map is well-formed and conserves the topology of the input. Moreover, the distortion declines and converges toward 0.0025, pointing out first that the loss of information during learning is low and that the structure in the self-organizing map is preserved. However, the boundary effects (the density of points is higher at the boundary of the map in panel (A)) affect both the distortion (it does not converge to zero; see panel (C) in Fig. 2) and the $\delta x - \delta y$ representation (it is not perfectly aligned with the red line; see panel (B) in Fig. 2). In spite of these boundary effects, the obtained $\delta x-\delta y$ performance indicator is good ($\mathcal{P}=0.01$).

The evolution of the norm-2 of feed-forward weights of three randomly chosen units ($r^{\ast }=(0.25, 0.25)$, $(0.1, 0.225)$, $(0.35, 0.075)$) is shown in the panel (D) of Fig. 2. This implies that the weights converge to an equilibrium after a transient period of about 2000 epochs. The oscillations around the equilibrium are due to a repeated alteration of the input stimulus, which causes a shift to the feed-forward weights values of each winner neuron (see [8] for more detail).

### Unstable case

**Figure 3:**
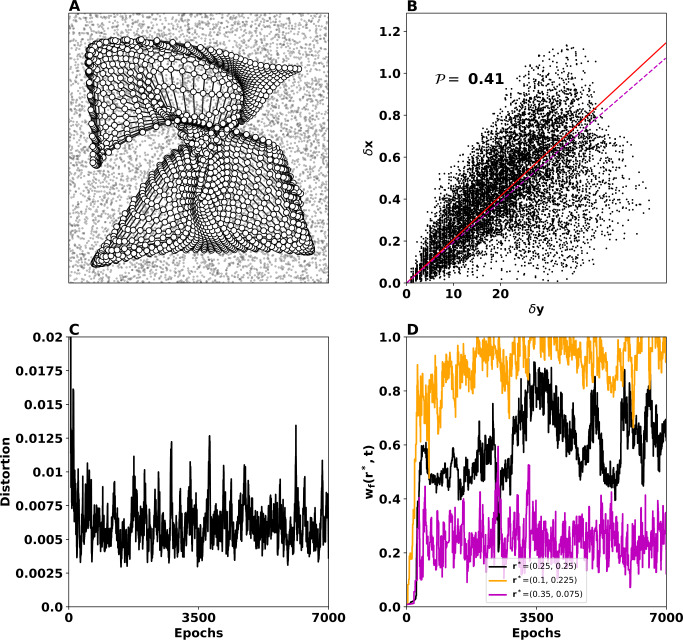
Two-dimensional SOM performance in the unstable case. (**A**) Feed-forward weights (white discs) as they have failed to organize into a topographic map after 7000 epochs. The input in this case is a two-dimensional rectangular uniform distribution (black dots). (**B**) $\delta x - \delta y$ representation (black cloud), mean of *δx* (red line), and the linear regression of the $\delta x - \delta y$ representation (magenta line). The fact that the cloud looks diffused indicates that the topographic map is not well organized, as confirmed by a high value of $\mathcal{P}=0.41$. (**C**) Distortion indicates that the loss of information during the learning process never drops (converges to an equilibrium) instead it oscillates. This means that the mapping of the input data to a two-dimensional map has failed. (**D**) Temporal evolution of norm-2 of feed-forward weights of three neurons placed at $r^{\ast }=(0.25, 0.25)$, $(0.1, 0.225)$, and $(0.35, 0.075)$). Condition (15) is violated, and accordingly the weights do not converge to an equilibrium

The second line of Table 1 provides parameters for which Condition (15) is violated ($5.25 > 1$). According to our theoretical predictions, the model might not be stable and thus may not be able to develop any self-organizing map at all. To make sure that this is the case (and not merely a transient effect), we have let the training take more epochs ($20{,} 000$). Nevertheless, here we present only the 7000 first epochs for consistency with the rest of our experiments. This situation is illustrated in Fig. 3, where the self-organizing process has failed to generate a well-formed map (panel (A)). In this case, it is apparent that self-organization process has failed to generate a topographic map.

The $\delta x - \delta y$ representation in panel (B) of Fig. 3 looks like a diffused cloud, indicating that there is no correlation between the grid points and the feed-forward weights, meaning that there is no preservation of the topology of the input space. Accordingly, the performance index reaches the value $\mathcal{P}=0.41$, thus higher than the stable case. Moreover, the distortion in panel (C) of Fig. 3 oscillates without converging to an equilibrium, pointing out that the loss of information remains high and therefore the mapping is not successful. Finally, the norm-2 of feed-forward weights of three units ($r^{\ast }=(0.25, 0.25)$, $(0.1, 0.225)$, $(0.35, 0.075)$) are shown in panel (D): it is apparent that they do not converge to an equilibrium. Instead, they oscillate violently and never stabilize around an equilibrium configuration.

### Numerical assessment of Corollary 2

**Figure 4:**
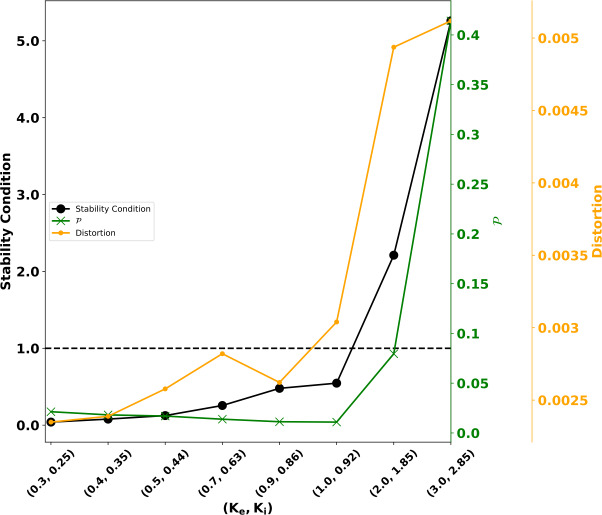
Numerical investigation of Corollary 2. Eight different pairs of the parameters $(K_{e}, K_{i})$ were used to investigate the conservativeness of the stability condition given by Corollary 2. We ran eight different simulations for 7000 epochs, keeping always the rest of the parameters as in Table 1 and the same PRNG seed as before (7659). The black curve indicates the numerical value of the left-hand side of (15): the stability is guaranteed if it is below the black dashed line. The green curve indicates the $\delta x - \delta y$ performance index $\mathcal{P}$. The orange curve represents the distortion $\mathcal{D}$ averaged of the 10 last epochs. It is apparent that as the values of $(K_{e}, K_{i})$ increase the Corollary 2 becomes violated and the self-organizing map fails to map the input space to the neural one (see Fig. 5 for more detail)

**Figure 5:**
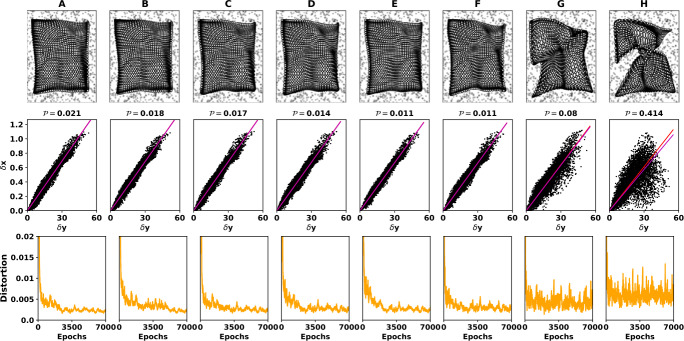
Numerical Investigation of Corollary 2. For the same eight experiments as in Fig. 4, the obtained self-organizing map is provided (first line), together with its $\delta x-\delta y$ representation (second line) and the evolution of the distortion (third line). The mean *δx* is represented as a red line, whereas the slope of the linear regression is given as a magenta line. (**A**): ($K_{e}=0.30$, $K_{i}=0.25$), (**B**): ($K_{e}=0.4$, $K_{i}=0.35$), (**C**): ($K_{e}=0.5$, $K_{i}=0.45$), (**D**): ($K_{e}=0.7$, $K_{i}=0.63$), (**E**): ($K_{e}=0.9$, $K_{i}=0.86$), (**F**): ($K_{e}=1.0$, $K_{i}=0.92$). In line with Fig. 4, a relevant map is obtained for the first five experiments (for which condition (15) is fulfilled), whereas for the two last self-organizing maps (**G**): ($K_{e}=2$, $K_{i}=1.85$) and (**H**): ($K_{e}=3$, $K_{i}=2.85$), the stability condition (15) is violated. This violation results in a nonstable neural field equation, and thus the self-organizing maps do not learn properly the representations

Finally, we numerically tested condition (15) of Corollary 2 for different values of the parameters $K_{e}$ and $K_{i}$ (all other parameters remained the same as in Table 1). For each pair $(K_{e}, K_{i})$, we computed the left-hand side of Eq. (15), the distortion $\mathcal{D}$ (averaged over the last 10 epochs), and the $\delta x - \delta y$ performance index $\mathcal{P}$; see Fig. 4. We observe that for high values of $K_{e}$ and $K_{i}$, the stability condition of Corollary 2 is violated (the black solid line overpasses the black dashed line). The distortion (orange curve) closely follows the left-hand side of condition (15) (up to a scaling factor), suggesting that distortion can serve as a measure of stability of system (1)–(5). Furthermore, the distortion and the $\delta x - \delta y$ performance index $\mathcal{P}$ indicate that the learning process degrades for high values of $(K_{e},K_{i})$, in line with the fact that condition (15) is violated. Figure 5 confirms this good alignment between the theoretical stability condition and the performance of the self-organizing map: for the first five cases, it properly maps the input space to the neural one, whereas the topology of the input space is not preserved in the last two cases, and a malformed topographic map is obtained.

## Conclusion

In this work, we have presented theoretical conditions for the stability of a neural field system coupled with an Oja-like learning rule [26]. Numerical assessments on a two-dimensional self-organizing map indicate that the theoretical condition is closely aligned with the capacity of the network to form a coherent topographic map.

Previous works have shown through simulations that the dynamical system described by Eqs. (1)–(5) can develop topographic maps through an unsupervised self-organization process [8, 9]. The model relies on the activity of a neural field to drive a learning process. This type of models are capable of developing topographic maps and reorganize them in face of several kinds of disturbances. Here we proceed to a rigorous theoretical analysis of such kind of models by employing neural field Lyapunov theory.

The obtained stability conditions are reminiscent of those obtained for general neural fields dynamics, in which the spatial $L_{2}$-norm of synaptic weights plays an essential role [11, 22, 33]. In our setting, these conditions translate in a good balance between excitation and inhibition for the exponential stability of the model equilibrium, thus allowing the self-organizing process to develop topographic maps. It is worth stressing that the proof techniques employed here do not rely on a linearization of the system around the considered equilibrium; it thus allows us to cover nondifferentiable activation functions (such as classical saturation or rectification functions).

These stability conditions provide a means to identify the parameters set within which the unsupervised learning works efficiently and thus provides an indication on how to tune them in practice. In particular, they can be used to further investigate how the dynamics of an underlying system affects the learning process during an unsupervised training process and what is the effect of the parameters on the final topographic map: as Fig. 4 indicates, the parameters of the model directly affect the quality of the topographic map. However, a limitation of the present work is that it does not offer a way of choosing the parameters in an optimal way. Furthermore, although the conditions provided by Theorem 1 guarantee the stability of the neural field, they do not predict the quality of the obtained map: Stability ensures that the learning will converge to an equilibrium, but the quality of the obtained map strongly depends on the structure of this equilibrium and hence on the chosen initial values of the feed-forward weights. This is a well-known problem with self-organizing maps [19], which is generally solved using a decreasing neighborhood, starting from a very wide one. In our case the neighborhood function is directly correlated with the profile of the field activity and is *fixed* (stereotyped). We thus cannot always ensure the proper unfolding of the map. It is to be noted that when the neighborhood of a Kohonen is kept fixed, it suffers from similar problems. Nevertheless, the numerical assessment of the proposed theoretical stability conditions suggests that the stability condition accurately predicts the emergence of topographic maps through unsupervised learning: see Figs. 4 and 5.

Other works have studied stability conditions for Kohonen maps and vector quantization algorithms using methods from linear systems stability theory [32] or through energy functions [10]. However, these works focus on the learning rule for the Kohonen self-organizing maps [20], and the dynamics are not explicitly given by dynamical systems. Our work goes beyond by taking into account not only the learning dynamics, but also the neural dynamics that drives the self-organizing process.

Last but not least, it has been shown that neural adaptation is crucial in the development of the neocortex [23] and neurons tend to adapt their input/output relation according to the statistics of the input stimuli. Our theoretical results provide conditions under which this input/output adaptation successfully takes place at least at a computational level.

## Proof of the theoretical results

### Proof of Theorem 1

To place the equilibrium at the origin, we employ the following change of variables:

$$\begin{aligned} &{\tilde{u}}(r, t)= u(r, t) - u^{*}(r), \\ &{\tilde{w}}_{f}(r, t)= w_{f}(r, t) - w_{f}^{*}(r), \end{aligned}$$

where $u^{*}$ and $w_{f}^{*}$ denote the equilibrium patterns of Eq. (6a) and (6b), as defined in Eq. (7a) and (7b). Then system (6a) and (6b) can be written as

17a $$\begin{aligned} &\tau \frac{\partial {\tilde{u}}}{\partial t}(r, t)= -{\tilde{u}}( r, t) + \int _{\Omega } w_{l}\bigl(r,r'\bigr)\tilde{f}_{l}\bigl(r', \tilde{u}\bigl(r',t\bigr)\bigr)\,dr' + \tilde{f}_{s}\bigl({\tilde{w}}_{f}(r, t)\bigr), \\ \end{aligned}$$

17b $$\begin{aligned} &\frac{\partial {\tilde{w}}_{f}}{\partial t}(r, t)= -\gamma { \tilde{w}}_{f}( r, t) \int _{\Omega } w_{e}\bigl(r,r'\bigr) \tilde{f}_{e}\bigl({\tilde{u}}\bigl(r',t\bigr)\bigr)\,dr', \end{aligned}$$

where for all $x\in \mathbb{R}$ and all $r\in \Omega $,

$$\begin{aligned} &\tilde{f}_{l}(r,x)=f_{l}\bigl(x+{u^{*}}( r)\bigr)- f_{l}\bigl({u^{*}}(r)\bigr), \\ &\tilde{f}_{s}(x)=f_{s}(x)-f_{s}(0), \\ &\tilde{f}_{e}(r,x)=f_{e}\bigl(x+{u^{*}}( r)\bigr). \end{aligned}$$

With this notation, we have $\tilde{f}_{l}(r,0)=\tilde{f}_{s}(0)=0$ for all $r\in \Omega $, meaning that (17a) and (17b) owns an equilibrium at zero. Thus the stability properties of the origin of (17a) and (17b) determine those of the equilibria of (6a) and (6b).

First, observe that since $w_{e}$ is a bounded function and Ω is compact, there exists $\bar{\omega }_{e}>0$ such that

18 $$\begin{aligned} \int _{\Omega }w_{e}\bigl(r,r' \bigr)^{2}\,dr'\leq \bar{w}_{e}^{2}, \quad \forall r \in \Omega . \end{aligned}$$

To assess the stability of (17a) and (17b), we may be tempted to rely on linearization techniques. Nevertheless, the linearized system (17a) and (17b) around the origin would necessarily involve the derivative of $f_{s}$ at zero, which may be undefined if $f_{s}$ is not differentiable at zero (which is the case for the system of interest (1)–(5), where $f_{s}$ involves an absolute value). Consequently, the proof we propose here relies on Lyapunov methods [17], which were extended to neural fields in [11].

Consider the following Lyapunov functional:

19 $$\begin{aligned} V(t) &:= \frac{\tau }{2} \int _{\Omega } {\tilde{u}}(r, t)^{2}\,dr+ \frac{\rho }{2\gamma } \int _{\Omega } {\tilde{w}}_{f}(r,t)^{2}\,dr, \end{aligned}$$

where $\rho >0$ denotes a parameter whose value will be decided later. First, observe that the following bounds hold at all $t\geq 0$:

20 $$\begin{aligned} \underline{\alpha } \bigl( \bigl\Vert \tilde{u}(\cdot ,t) \bigr\Vert ^{2}+ \bigl\Vert \tilde{w}_{f}( \cdot ,t) \bigr\Vert ^{2} \bigr)\leq V(t)\leq \overline{\alpha } \bigl( \bigl\Vert \tilde{u}(\cdot ,t) \bigr\Vert ^{2}+ \bigl\Vert \tilde{w}_{f}(\cdot ,t) \bigr\Vert ^{2} \bigr), \end{aligned}$$

where $\underline{\alpha }:=\frac{1}{2}\min \{\tau ; \rho /\gamma \}>0$ and $\overline{\alpha }:=\frac{1}{2}\max \{\tau ; \rho /\gamma \}>0$. The derivative of *V* along the solutions of (6a) and (6b) reads

21 $$\begin{aligned} \dot{V}(t) ={}& \tau \int _{\Omega } {\tilde{u}}(r,t) \frac{\partial {\tilde{u}}(r,t)}{\partial t}\,dr+ \frac{\rho }{\gamma } \int _{\Omega }{\tilde{w}}_{f}(r,t) \frac{\partial {\tilde{w}}_{f}(r,t)}{\partial t}\,dr \\ ={}& \int _{\Omega } {\tilde{u}}(r,t) \biggl[ -{\tilde{u}}(r,t) + \int _{\Omega }w_{l}\bigl(r,r'\bigr) \tilde{f}_{l}\bigl(r', \tilde{u}\bigl( r',t\bigr)\bigr)\,dr' + \tilde{f}_{s}\bigl({\tilde{w}}_{f}(r,t)\bigr) \biggr]\,dr \\ &{}- \rho \int _{\Omega } \biggl[{\tilde{w}}_{f}(r, t)^{2} \int _{\Omega }w_{e}\bigl(r,r'\bigr)\tilde{f}_{e}\bigl(r', \tilde{u}\bigl(r',t\bigr)\bigr)\,dr' \biggr]\,dr. \end{aligned}$$

Moreover, denoting by $\ell _{s}$, $\ell _{e}$, and $\ell _{l}$ the Lipschitz constants of $f_{s}$, $f_{e}$, and $f_{l}$ respectively, we have that for all $x\in \mathbb{R}$ and all $r\in \Omega $.

22 $$\begin{aligned} &\bigl\vert \tilde{f}_{l}(r,x) \bigr\vert \leq \ell _{l} \vert x \vert , \end{aligned}$$

23 $$\begin{aligned} &\bigl\vert \tilde{f}_{s}(x) \bigr\vert \leq \ell _{s} \vert x \vert , \end{aligned}$$

24 $$\begin{aligned} &\bigl\vert \tilde{f}_{e}(r,x)-\tilde{f}_{e}( r,0) \bigr\vert \leq \ell _{e} \vert x \vert . \end{aligned}$$

Applying the Cauchy–Schwarz inequality and using Eq. (22), it follows that

$$\begin{aligned} \int _{\Omega }w_{l}\bigl(r,r'\bigr) \tilde{f}_{l}\bigl(r', \tilde{u}\bigl( r',t\bigr)\bigr)\,dr' & \leq \int _{\Omega } \bigl\vert w_{l}\bigl(r, r'\bigr) \bigr\vert \bigl\vert \tilde{f}_{l} \bigl(r',\tilde{u}\bigl(r',t\bigr)\bigr) \bigr\vert \,dr' \\ &\leq \ell _{l} \int _{\Omega } \bigl\vert w_{l}\bigl(r, r'\bigr) \bigr\vert \bigl\vert \tilde{u}\bigl( r',t\bigr) \bigr\vert \,dr' \\ &\leq \ell _{l} \sqrt{ \int _{\Omega }w_{l}\bigl(r,r'\bigr)^{2}\,dr'}\sqrt{ \int _{\Omega }\tilde{u}\bigl(r',t \bigr)^{2}\,dr'}. \end{aligned}$$

Hence, using again the Cauchy–Schwarz inequality,

$$\begin{aligned} &\int _{\Omega }\tilde{u}(r,t) \biggl[ \int _{\Omega }w_{l}\bigl(r, r'\bigr) \tilde{f}_{l}\bigl(r', \tilde{u}\bigl(r',t\bigr)\bigr)\,dr' \biggr]\,dr\\ &\quad \leq \ell _{l} \int _{\Omega } \bigl\vert \tilde{u}(r,t) \bigr\vert \biggl[ \sqrt{ \int _{ \Omega }w_{l}\bigl(r,r'\bigr)^{2}\,dr'}\sqrt{ \int _{\Omega }\tilde{u}\bigl(r',t \bigr)^{2}\,dr'} \biggr]\,dr\\ &\quad \leq \ell _{l}\sqrt{ \int _{\Omega }\tilde{u}(r,t)^{2}\,dr} \sqrt{ \int _{\Omega } \biggl[ \int _{\Omega }w_{l}\bigl(r,r'\bigr)^{2}\,dr' \int _{\Omega }\tilde{u}\bigl(r',t \bigr)^{2}\,dr' \biggr]\,dr}. \end{aligned}$$

Observing that $\int _{\Omega }\tilde{u}(r',t)^{2}\,dr'$ is independent of *r* and defining

25 $$\begin{aligned} \bar{w}_{l}:=\sqrt{ \int _{\Omega } \int _{\Omega }w_{l}\bigl(r,r'\bigr)^{2}\,dr'\,dr}, \end{aligned}$$

it follows that

26 $$\begin{aligned} \int _{\Omega } \tilde{u}(r,t) \biggl[ \int _{\Omega }w_{l}\bigl(r, r'\bigr) \tilde{f}_{l}\bigl(r', \tilde{u}\bigl(r',t\bigr)\bigr)\,dr' \biggr]\,dr\leq \ell _{l}\bar{w}_{l} \int _{\Omega }\tilde{u}(r,t)^{2}\,dr. \end{aligned}$$

Furthermore, using Eq. (23), we have that

$$\begin{aligned} \int _{\Omega }\tilde{u}(r,t)\tilde{f}_{s} \bigl(w_{f}(r,t)\bigr)\,dr& \leq \ell _{s} \int _{\Omega } \bigl\vert \tilde{u}(r,t) \bigr\vert \bigl\vert w_{f}( r,t) \bigr\vert \,dr\\ &\leq \ell _{s}\sqrt{ \int _{\Omega }\tilde{u}(r,t)^{2}\,dr} \sqrt{ \int _{\Omega }w_{f}(r,t)^{2}\,dr}. \end{aligned}$$

Invoking the inequality $2ab \leq (a^{2}/\lambda + \lambda b^{2})$ for all $a, b \in \mathbb{R}$ and $\lambda > 0$, we obtain that

27 $$\begin{aligned} \int _{\Omega }\tilde{u}(r,t)\tilde{f}_{s} \bigl(\tilde{w}_{f}(r,t)\bigr)\,dr\leq \frac{\ell _{s}}{2} \biggl(\lambda \int _{\Omega }\tilde{u}( r,t)^{2}\,dr+ \frac{1}{\lambda } \int _{\Omega }\tilde{w}_{f}( r,t)^{2}\,dr\biggr) \end{aligned}$$

for any $\lambda >0$.

Now assumption (10) ensures that $\inf_{r\in \Omega }\int _{\Omega }w_{e}(r,r') \tilde{f}_{e}(r',0)\,dr' >0$. It follows that there exists $c>0$ such that

$$\begin{aligned} \int _{\Omega }w_{e}\bigl(r,r'\bigr)\tilde{f}_{e}\bigl(r',0 \bigr)\,dr' \geq 2c, \quad \forall r\in \Omega . \end{aligned}$$

Consequently, using (24) and the Cauchy–Schwarz inequality, we get that for any $v\in L_{2}(\Omega ,\mathbb{R})$,

$$\begin{aligned} &\int _{\Omega }w_{e}\bigl(r,r'\bigr)\tilde{f}_{e}\bigl(r',v \bigl(r'\bigr)\bigr)\,dr' \\ &\quad = \int _{\Omega }w_{e}\bigl(r,r'\bigr)\tilde{f}_{e}\bigl(r',0 \bigr)\,dr'+ \int _{\Omega }w_{e}\bigl(r,r'\bigr) \bigl(\tilde{f}_{e}\bigl( r',v\bigl(r'\bigr)\bigr)-\tilde{f}_{e} \bigl(r',0\bigr) \bigr)\,dr' \\ &\quad \geq 2c- \int _{\Omega } \bigl\vert w_{e}\bigl(r, r'\bigr) \bigr\vert \bigl\vert \tilde{f}_{e} \bigl(r',v\bigl(r'\bigr)\bigr)- \tilde{f}_{e}\bigl(r',0\bigr) \bigr\vert d r' \\ &\quad \geq 2c-\ell _{e} \int _{\Omega } \bigl\vert w_{e}\bigl(r, r'\bigr) \bigr\vert \bigl\vert v\bigl(r'\bigr) \bigr\vert \,dr' \\ &\quad \geq 2c-\ell _{e}\sqrt{ \int _{\Omega }w_{e}\bigl(r,r'\bigr)^{2}\,dr'}\sqrt{ \int _{\Omega }v\bigl(r'\bigr)^{2}\,dr'} \\ &\quad \geq 2c-\ell _{e}\bar{w}_{e} \Vert v \Vert , \end{aligned}$$

where the last bound comes from (18). Let $\mathcal{B}_{\varepsilon }$ denote the ball (in $L_{2}$-norm) of radius $\varepsilon >0$, that is, $\mathcal{B}_{\varepsilon }:= \{ v\in L_{2}(\Omega ,\mathbb{R}) : \|v\|< \varepsilon \} $. Letting $\varepsilon := c/\ell _{e}\bar{w}_{e}$, we conclude from the above expression that

28 $$\begin{aligned} \int _{\Omega }w_{e}\bigl(r,r'\bigr)\tilde{f}_{e}\bigl(r',v \bigl(r'\bigr)\bigr)\,dr'\geq c,\quad \forall r\in \Omega , \forall v\in \mathcal{B}_{\varepsilon }. \end{aligned}$$

Consider an initial condition such that $\tilde{u}(\cdot ,0)\in \mathcal{B}_{\varepsilon }$ and let $T\in [0,+\infty ]$ denote the time needed for $\tilde{u}(\cdot ,t)$ to leave $\mathcal{B}_{\varepsilon }$. Then, by definition, $\tilde{u}(\cdot ,t)\in \mathcal{B}_{\varepsilon }$ for all $t\in [0,T)$, and $\tilde{u}(\cdot ,T)\notin \mathcal{B}_{\varepsilon }$ if *T* is finite. Note that by the continuity of solutions, $T>0$. Moreover, in view of (28),

29 $$\begin{aligned} \int _{\Omega }w_{e}\bigl(r,r'\bigr)\tilde{f}_{e}\bigl(r', \tilde{u}\bigl( r',t\bigr)\bigr)\,dr'\geq c,\quad \forall t\in [0,T),\forall r\in \Omega . \end{aligned}$$

Combining Eqs. (21), (22), (23), and (29), we obtain that for all $t\in [0,T)$,

$$\begin{aligned} \dot{V}(t) &\leq - \biggl(1-\ell _{l}\bar{w}_{l}- \frac{\lambda \ell _{s}}{2} \biggr) \int _{\Omega } {\tilde{u}}(r,t)^{2}\,dr- \biggl(\rho c-\frac{\ell _{s}}{2\lambda } \biggr) \int _{\Omega }\tilde{w}_{f}(r,t)^{2}\,dr \end{aligned}$$

Pick $\lambda =(1-\ell _{l}\bar{w}_{l})/\ell _{s}$. Note that $\lambda >0$ since $\ell _{l}\bar{w}_{l}<1$ by assumption (see Eq. (9)). Then the choice $\rho =\frac{\ell _{s}}{c\lambda }= \frac{\ell _{s}^{2}}{c(1-\ell _{l}\bar{w}_{l})}>0$ leads to

$$\begin{aligned} \dot{V}(t) &\leq -\frac{1}{2} \bigl\Vert {\tilde{u}}(\cdot ,t) \bigr\Vert ^{2} - \frac{\rho c}{2} \bigl\Vert \tilde{w}_{f}(\cdot ,t) \bigr\Vert ^{2} \\ &\leq -\frac{1}{2}\min \{1 ; \rho c\} \bigl( \bigl\Vert {\tilde{u}}( \cdot ,t) \bigr\Vert ^{2} + \bigl\Vert \tilde{w}_{f}( \cdot ,t) \bigr\Vert ^{2} \bigr). \end{aligned}$$

Using (20) and letting $\alpha :=\frac{1}{2\overline{\alpha }}\min \{1 ; \rho c\}>0$, we finally obtain that

$$\begin{aligned} \dot{V}(t)\leq -\alpha V(t), \quad \forall t\in [0,T). \end{aligned}$$

Integrating this gives $V(t)\leq V(0)e^{-\alpha t}$ for all $t\in [0,T)$, which yields, using (20),

30 $$\begin{aligned} \bigl\Vert \tilde{u}(\cdot ,t) \bigr\Vert ^{2}+ \bigl\Vert \tilde{w}_{f}(\cdot ,t) \bigr\Vert ^{2} \leq \frac{\overline{\alpha }}{\underline{\alpha }} \bigl( \bigl\Vert \tilde{u}(\cdot ,0) \bigr\Vert ^{2}+ \bigl\Vert \tilde{w}_{f}(\cdot ,0) \bigr\Vert ^{2} \bigr)e^{-\alpha t},\quad \forall t \in [0,T). \end{aligned}$$

Thus if initial conditions are picked within the $L_{2}$-ball of radius $\frac{\sqrt{\underline{\alpha }}}{\varepsilon \sqrt{\overline{\alpha }}}$, then $\|\tilde{u}(\cdot ,t)\|+\|\tilde{w}_{f}(\cdot ,t)\|< \varepsilon $ at all times $t\geq 0$. This means that for these initial conditions, solutions never leave the ball $\mathcal{B}_{\varepsilon }$, and hence $T=+\infty $. Thus Eq. (30) ensures the exponential stability on this set of initial conditions.

### Proof of Corollary 1

Assumption described by Eq. (13) is equivalent to requiring $\bar{w}_{l}<1$, with $\bar{w}_{l}$ defined in Eq. (25). Since the Lipschitz constant of the rectification is $\ell _{l}=1$, this makes Eq. (9) fulfilled.

Moreover, we claim that the solution $u^{*}$ of the implicit Eq. (12a) and (12b) is necessarily positive on some subset of Ω of nonzero measure. To see this, assume on the contrary that $u^{*}(r)\leq 0$ for almost all $r\in \Omega $. Then $\operatorname{rect}(u^{*}(r))=0$ for almost all $r\in \Omega $, which implies that

$$ \int _{\Omega } w_{l}\bigl(r,r'\bigr) \operatorname{rect}\bigl(u^{*}\bigl(r'\bigr)\bigr)\,dr' =0, \quad \forall r \in \Omega . $$

In view of Eq. (12a) and (12b), this implies that $u^{*}(r)=1$ for all $r\in \Omega $, thus leading to a contradiction. Consequently, as claimed, $u^{*}$ is necessarily positive on some subset $\Omega ^{+}$ of Ω of nonzero measure. Recalling that here Ω is assumed to be a compact set, it follows that

$$ \inf_{r\in \Omega } \int _{\Omega }e^{-|r-r'|^{2}/2\sigma _{e}^{2}} \operatorname{rect}\bigl(u^{*}(r) \bigr)\,dr \geq \inf_{r\in \Omega } \int _{\Omega ^{+}} e^{-|r-r'|^{2}/2 \sigma _{e}^{2}} u^{*}(r)\,dr>0, $$

which makes Eq. (10) satisfied. The conclusion then follows from Theorem 1.

### Proof of Corollary 2

The following one-dimensional relation holds:

31 $$\begin{aligned} \int _{a}^{b} \int _{a}^{b} e^{-\frac{|x-y|^{2}}{2\sigma ^{2}}}\,dx\,dy= \sqrt{\xi _{a,b}(\sigma )}. \end{aligned}$$

To compute its two-dimensional counterpart, let $r=(r_{1},r_{2})$ and $r'=(r_{1}',r_{2}')$. Then, for $\Omega =[a,b]\times [a,b]$,

$$\begin{aligned} \int _{\Omega } \int _{\Omega }e^{-\frac{|r-r'|^{2}}{2\sigma ^{2}}}\,dr'\,dr&= \int _{a}^{b} \int _{a}^{b} \int _{a}^{b} \int _{a}^{b} \exp \biggl(- \frac{(r_{1}-r_{1}')^{2}+(r_{2}-r_{2}')^{2}}{2\sigma ^{2}} \biggr)\,dr_{1}'\,dr_{2}'\,dr_{1}\,dr_{2}. \end{aligned}$$

Using Fubini’s theorem, it follows that

$$\begin{aligned} &\int _{\Omega } \int _{\Omega }e^{-\frac{|r-r'|^{2}}{2\sigma ^{2}}}\,dr'\,dr \\ &\quad = \int _{a}^{b} \int _{a}^{b}\exp \biggl(- \frac{(r_{1}-r_{1}')^{2}}{2\sigma ^{2}} \biggr) \biggl( \int _{a}^{b} \int _{a}^{b} \exp \biggl(- \frac{(r_{2}-r_{2}')^{2}}{2\sigma ^{2}} \biggr)\,dr_{2}\,dr_{2}' \biggr)\,dr_{1}\,dr_{1}'. \end{aligned}$$

By Eq. (31) this gives

32 $$\begin{aligned} \int _{\Omega } \int _{\Omega }e^{-\frac{|r-r'|^{2}}{2\sigma ^{2}}}\,dr'\,dr &= \int _{a}^{b} \int _{a}^{b}\exp \biggl(- \frac{(r_{1}-r_{1}')^{2}}{2\sigma ^{2}} \biggr)\sqrt{\xi _{a,b}( \sigma )}\,dr_{1}\,dr_{1}'. \\ &=\xi _{a,b}(\sigma ). \end{aligned}$$

The left-hand term of Eq. (13) then reads

$$\begin{aligned} &\int _{\Omega } \int _{\Omega } \bigl(K_{e}e^{- \frac{|r-r'|^{2}}{2\sigma _{e}^{2}}}-K_{i}e^{- \frac{|r-r'|^{2}}{2\sigma _{i}^{2}}} \bigr)^{2}\,dr'\,dr \\ &\quad = \int _{\Omega } \int _{\Omega } \bigl(K_{e}^{2}e^{- \frac{|r-r'|^{2}}{\sigma _{e}^{2}}}+K_{i}^{2}e^{- \frac{|r-r'|^{2}}{\sigma _{i}^{2}}}-2K_{e}K_{i}e^{- \frac{|r-r'|^{2}\sigma _{e}\sigma _{i}}{2\sqrt{\sigma _{e}^{2}+\sigma _{i}^{2}}}} \bigr)\,dr'\,dr \\ &\quad =K_{e}^{2}\xi _{a,b}(\sigma _{e}/\sqrt{2})+K_{i}^{2}\xi _{a,b}( \sigma _{i}/\sqrt{2})-2K_{e}K_{i} \xi _{a,b} \biggl( \frac{\sigma _{e}\sigma _{i}}{\sqrt{\sigma _{e}^{2}+\sigma _{i}^{2}}} \biggr), \end{aligned}$$

which concludes the proof.

## PRNG seed

We ran both stable and nonstable experiments ten times with different PRNG seeds. All the PRNG seeds we used are 10, 74, 433, 721, 977, 1330, 3433, 5677, 9127, 7659.

## Data Availability

The source code used in this work for running the simulations, analyzing the results, and plotting the figures is freely distributed under the GPL-3 License and can be found at https://github.com/gdetor/som_stability.

## Abbreviations

SOM: Self-organizing map
DSOM: Dynamic self-organizing map
FFT: Fast Fourier transform
PRNG: Pseudorandom number generator
